# What the Insurance World Say

**Published:** 1888-02-11

**Authors:** 


### WHAT THE INSURANCE WORLD SAY.

The actual details in connexion with the establishment of the Fund have been well entrusted to Mr. George King. In a recent letter in the *Times*, Mr. King explains the benefits of the Fund to be twofold, including sickness relief and pensions in old age. As a protection to the Fund, no nurse will be allowed to effect a sickness policy without subscribing, in definite proportions, to the Pension Fund. In this way a hedge is obtained against any abnormal mortality; for, as Mr. King states, "if there is much sickness and a high rate of mortality, the Pension Fund will benefit; whereas, if the nurses should prove a healthy class, and the mortality be light, the Sickness Fund will benefit." In regard to the pensions, the recent Government annuitant experience has been adopted; and in this we can fully agree with Mr. King that an abundantly strong basis has been provided. It is estimated that if each nurse contributes one-eighth of her wages as an annual premium, the retiring allowance will not be less than 10*s.* per week. The payments will seem proportionately heavy to some; but substantial benefits cannot be secured without proper contributors. The management of the Fund is to be in the hands of a council consisting of members conversant with financial affairs; and it is probable that their services will be volunteered gratuitously.—*The Post Magazine and Insurance Monitor.*

